# 

**Published:** 1856-10-01

**Authors:** 


### NOTICE.

Part I. of Dr. Webster's Notes on Belgian Lunatic Asylums, including the Insane Colony of Gheel, will appear in our next number.

### ASSOCIATION OF MEDICAL OFFICERS

OF

### ASYLUMS AND HOSPITALS FOR THE INSANE.

---

*Office-bearers and Committee of Management.*

*President*—Dr. Hitchman, Medical Superintendent, County Asylum, Derby.
*President Elect*—Dr. Forbes Winslow, D.C.L., Cavendish Square, London.
*Ex-President*—Dr. Thurnam, Medical Superintendent, County Asylum, Wilts.
*Treasurer*—W. Ley, Esq., Medical Superintendent, County Asylum, Oxfordshire.
*Auditors*—Dr. Campbell, Medical Superintendent, County Asylum, Essex; Dr. Prichard, Abingdon Abbey, Northampton.
*Hon. Secretary* (*General*)—Dr. L. Robertson, Charles Street, Berkeley Square, London.
*Hon. Secretary for Ireland*—Dr. Stewart, Medical Superintendent, District Asylum, Belfast.
*Hon. Secretary for Scotland*—Dr. Browne, Medical Superintendent, Royal Crichton Asylum, Dumfries.
*Editor of Journal*—Dr. Bucknill, Medical Superintendent, County Asylum, Devon.

*Other Members.*

Alderson, J. S., Esq., Medical Superintendent, West Riding County Asylum, Wakefield.
Allen, Dr., Medical Superintendent, County Asylum, South Wales.
Allen, Thomas, Esq., Medical Superintendent, Warneford Asylum, Oxford.
Arlidge, Dr., late Medical Superintendent, St. Luke's.
Armstrong, Dr., Peckham House, London.
Atkinson, J., Esq., Heyworth, York.
Bakewell, Dr., Church Stretton, Salop.
Berkeley, Dr., Medical Superintendent, District Asylum, Mullingar.
Berrow, William, Esq., Duddeston Hall, Birmingham.
Blount, J. H., Esq., M.B., Edgbaston, Birmingham.
Bodington, G., Esq., Driffold House, Sutton Coldfield, Warwickshire.
Boisragon, Dr., Medical Superintendent, County Asylum, Cornwall.
Boyd, Dr., Medical Superintendent, County Asylum, Somerset.
Brushfield, T. N., Esq., Medical Superintendent, County Asylum, Chester.
Buck, J., Esq., Medical Superintendent, County Asylum, Leicestershire.
Burnet, Dr., Westbroke House, Alton, Hants.
Bush, Dr., late of Sandywell Park, Gloucestershire.
Bush, J., Esq., Clapham Retreat, London.
Casson, Edward, Esq., Medical Superintendent, Norfolk County Asylum, near Norwich.
Casson, F. W., Esq., Medical Superintendent, Hull Borough Asylum.
Chapman, Dr., County Asylum, Wilts.
Chevalier, Dr., The Grove, Ipswich.
Chewner, Dr., Visiting Physician, Lincoln Hospital for the Insane.
Cleaton, J., Esq., Medical Superintendent, County Asylum, Rainhill.
Cole, Henry, Esq., Dartmouth House, Lewisham, Kent.
Conolly, Dr., D.C.L., Hanwell, Middlesex.
Corbett, Dr., Medical Superintendent, State Asylum, Dundrum.
Cornwall, James, Esq., Fairford, Gloucestershire.

Corsellis, Dr., late Medical Superintendent, County Asylum, Wakefield.
Dalrymple, D., Esq., Heigham Retreat, Norwich.
Daniel, Dr., Sillwood, Brighton.
Davey, Dr., Northwoods, Bristol.
Diamond, Dr., Medical Superintendent, County Asylum, Surrey.
Dickson, Dr., Medical Superintendent, Royal Asylum, Cheadle.
Eccleston, T., Esq., late Medical Superintendent, County Asylum, Liverpool.
Eddison, Booth, Esq., Nottingham.
Fayrer, Dr., Henley-in-Arden.
Foote, Dr., late Medical Superintendent, County Asylum, Norfolk.
Formby, Dr., Liverpool.
Flynn, Dr., Medical Superintendent, District Asylum, Clonmel.
Freer, W., Esq., Duddeston Hall, Birmingham.
Green, Thomas, Esq., Medical Superintendent, Borough Asylum, Birmingham.
Harrison, Professor, Dublin.
Hastings, Sir Charles, D.C.L., Worcester.
Hedger, Dr., County Asylum, Colney Hatch, Middlesex.
Helps, Dr., Royal Bethlem Hospital.
Hervey, Dr., Assistant Medical Officer, Haslar Naval Hospital, Portsmouth.
Hewson, Dr., Medical Superintendent, Coton Hill Asylum, Stafford.
Hill, R. G., Esq., Inverness Lodge, and Wyke House, Brentford.
Hills, W. C., Esq., County Asylum, Kent.
Hitch, Dr., Sandywell Park, Gloucestershire.
Hitchcock, C., Esq., Market Lavington, Wilts.
Hood, Dr., Resident Physician, Royal Hospital of Bethlem.
Horsbrugh, Dr. Norwood Green, Middlesex.
Huxley, Dr., Medical Superintendent, County Asylum, Kent.
Iles, Albert, Esq., Cirencester, Gloucestershire.
Jones, G. T., Esq , Medical Superintendent, County Asylum, North Wales.
Kirkman, W. P., Esq., County Asylum, Devon.
Kirkman, Dr., Medical Superintendent, County Asylum, Suffolk.
Kitching, J., Esq., Medical Superintendent, The Retreat, York.
Langworthy, R., Esq., Plympton House, Devon.
Lawlor, Dr., Medical Superintendent, Killarney District Asylum.
Little, Dr., Medical Superintendent, District Asylum, Sligo.
Lowry, Dr., West Malling, Kent.
Lynch, Dr., Sandfield House, Lichfield.
Mackintosh, Dr., Med. Superintendent, Royal Asylum, Gartnavel, Glasgow.
Mackintosh, Dr., Dinsdale Park, Darlington.
Macreight, Dr., Somerset County Asylum, Wells.
Mallam, R., Esq., Hook Norton, Oxon.
Manley, Dr., Medical Superintendent, County Asylum, Hants.
Marshall, W. G., Esq., Med. Superintendent, County Asylum, Colney Hatch.
Maxwell, Dr., Medical Superintendent, Asylum for Idiots, Redhill, Surrey.
Metcalfe, J. W., Esq., Accomb House, York.
Millard, G., Esq., Borough Asylum, Haverfordwest.
Millar, J. N., Esq., Medical Superintendent, County Asylum, Bucks.
Monro, Dr. H., 87, Harley-street, Visiting Physician, St. Luke's, London.
Morison, Sir Alexander, M.D., 6, Durham Terrace, Westbourne Park.
Muirhead, Dr., Longdales Asylum, Lanark.
Nesbit, Dr., Medical Superintendent, Hospital for the Insane, Northampton.
Niven, Dr., H.E.I.C. Service, late County Asylum, Essex.
Noble, Dr., Manchester.
Norton, Dr., Amroth Castle, South Wales.
Oliver, Dr., Medical Superintendent, County Asylum, Shropshire.
Palmer, Dr., Medical Superintendent, County Asylum, Lincolnshire.

Parsey, Dr., Medical Superintendent, County Asylum, Warwickshire.
Paul, J. H., Esq., Camberwell House, London.
Philp, Dr., late Visiting Physician, St. Luke's.
Power, Dr., Medical Superintendent, District Asylum, Cork.
Prosser, J., Esq., late Medical Superintendent, County Asylum, Leicester.
Robertson, J. G., Esq., County Asylum, Brentwood, Essex.
Sankey, H., Esq., County Asylum, Oxford.
Sankey, Dr., Medical Superintendent, County Asylum, Hanwell, Middlesex.
Seaton, Dr., Halliford House, Sunbury, Middlesex.
Shapter, Dr., Visiting Physician, St. Thomas' Hospital, Exeter.
Sherlock, Dr., Medical Superintendent, County Asylum, Worcester.
Simpson, Dr., Visiting Physician, York County Asylum.
Smith, J., Esq., Hadham Palace, Herts.
Smith, Dr., Park Place, Leeds.
Snape, C., Esq., Medical Superintendent, County Asylum, Surrey.
Stevens, Dr., Medical Superintendent, St. Luke's, London.
Stewart, Dr., Southall Park, Middlesex.
Stuart, Dr., Staff Surgeon, R.N., Lunatic Department, Haslar Hospital.
Stiff, Dr., Medical Superintendent, County Asylum, Nottingham.
Stillwell, G., Esq., Epsom, Surrey.
Sutherland, Dr., Richmond Terrace, Whitehall, Visiting Physician, St. Luke's, London.
Symes, J. G., Esq., Medical Superintendent, County Asylum, Dorset.
Terry, J., Esq., Bailbrook House, Bath.
Thompson, Dr., Belfast.
Thornton, Dr., Visiting Physician, District Asylum, Ballinasloe.
Tuke, Dr., The Retreat, York.
Tuke, Dr., Manor House, Chiswick.
Tyerman, F. D., Esq., Medical Superintendent, County Asylum, Colney Hatch.
De Vitre, Dr., Visiting Physician, County Asylum, Lancaster.
Walsh, F. D., Esq., Medical Superintendent, Hospital for the Insane, Lincoln.
Watson, J. F., Esq., Heigham Hall, Norwich.
Warwick, J., Esq., Laverstock House, Salisbury.
West, Dr., Medical Superintendent, District Asylum, Omagh.
Willett, Dr., Wyke House, Brentford.
Williams, Dr. L., Visiting Physician, County Asylum, Denbigh.
Williams, Caleb, Esq., York.
Williams, Dr., Medical Superintendent, County Asylum, Gloucester.
Wilson, R., Esq., County Asylum, Prestwich.
Wing, Dr., Longwoods House, Bristol.
Wingett, Dr., Medical Superintendent, Royal Asylum, Dundee.
Wood, Dr. W., Kensington House.

### *Honorary Members.*

Brodie, Sir Benjamin Collins, Bart., D.C.L., Saville Row.
Gaskell, S., Esq., Commissioner in Lunacy.
Holland, Sir Henry, Bart., M.D., D.C.L., 25, Brook Street, Grosvenor Square.
Hume, Dr., Commissioner in Lunacy.
Nugent, Dr., Inspector of Asylums, Ireland.
Peach, Dr., J. P., Langley Hall, Derby.
Tuke, Samuel, Esq., York.
White, Dr., Inspector of Asylum, Ireland.
Wilkes, J., Esq., Commissioner in Lunacy.

### ASSOCIATION OF MEDICAL OFFICERS

OF

### ASYLUMS AND HOSPITALS FOR THE INSANE.

At the Annual Meeting held at the County Asylum, Michael-over, near Derby, on the 1st of August, under the Presidency of Dr. Hitchman, the following Office-bearers were appointed for the year 1856-7:—

*President*—Dr. Hitchman, Medical Superintendent, County Asylum, Derby.
*President Elect*—Dr. Forbes Winslow, D.C.L., Cavendish Square, London.
*Ex-President*—Dr. Thurnam, Medical Superintendent, County Asylum, Wilts.
*Treasurer*—W. Ley, Esq., Medical Superintendent, County Asylum, Oxfordsh.
*Auditors*—Dr. Campbell, Medical Superintendent, County Asylum, Essex; Dr. Prichard, Abingdon Abbey, Northampton.
*Hon. Secretary* (*General*)—Dr. Lockhart Robertson, Charles-street, Berkeley-square, London.
*Hon. Secretary for Ireland*—Dr. Stewart, Medical Superintendent, District Asylum, Belfast.
*Hon. Secretary for Scotland*—Dr. Browne, Medical Superintendent, Royal Crichton Asylum, Dumfries.
*Editor of Journal*—Dr. Bucknill, Medical Superintendent, County Asylum, Devon.

The following Members were duly elected in accordance with the terms of Rules III. and VI.:—

*Honorary:—*

Sir Benjamin C. Brodie, Bart., D.C.L.
Sir Henry Holland, Bart., M.D., D.C.L.
James Wilkes, Esq., Her Majesty's Commissioner in Lunacy.
Thomas Peach, Esq., M.D., Justice of the Peace for the County of Derby.
J. R. Hume, Esq., M.D., Her Majesty's Commissioner in Lunacy.

*Ordinary:—*

Dr. Helps; Dr. Fayrer; Dr. Bakewill; F. W. Casson, Esq.; J. S. Alderson, Esq.; J. G. Robertson, Esq.; Dr. Willett; Dr. Hedger; T. Allen, Esq.; J. Bush, Esq.; Dr. Armstrong; Dr. Stewart; Dr. Macreight; Dr. Blount; Dr. Chewner; Dr. Boisragon; Dr. Hervey; G. Bodington, Esq.; Dr. Lawlor.

Members are reminded that by Rule IV., "the Annual Subscription of £1 1s. is due in advance on the 1st of July in each year."

It is particularly desired that the arrears for 1855-6 may be paid up without further delay.

All Subscriptions to be paid to the Treasurer, William Ley, Esq., Medical Superintendent, County Asylum, Oxford.

C. LOCKHART ROBERTSON, M.B. Cantab.
*Honorary Secretary.*

1, Charles Street, Berkeley Square,
September, 1856.

### To Correspondents.

Our Foreign Abstract of Psychological Literature will appear in the next Number.

Case of Mr. Snape.—In this case the grand jury ignored the bill of indictment, and of course all proceedings in the matter have ceased.

Vacant Appointments.—Cornwall County Lunatic Asylum, caused by the resignation of Dr. Boisragon; and County Lunatic Asylum, Bucks.

### INDEX TO VOL. IX.

A.

ABERDEEN, Royal Asylum of, 352
Anatomy and psychiatry, by Dr. F. W. Hagen, 465
Apparitions and dreams, physiological and psychological phenomena of, 373
Association of Medical Cfficers of Asylums and Hospitals for the Insane, annual meeting of, 646
Autobiography of the insane, 66, 202
Automatism, theory of, by M. Baillarger, 299

B.

Baillarger, M., on a medico-legal case of monomania, 101
———— on the theory of automatism, 299
Baily, Samuel, letters on the philosophy of the human mind, by, xiii
Beck, Dr. T. Romeyn, death of, iv
Blacks, abject condition of, 177
Bodily disease produced by lunar influence, 150
Brewster, Sir D., on spirit rapping, iii
Brain, its condition in somnambulism, 36
Brierre de Boismont, M., on monomania, 516
——— on suicide, 445

C.

Capital punishment, xxv, lxxxi
Change produced by war, 12
Children, suicide of, 296
Chloroform, hallucinations produced by, v
Coma, partial, with faintness, considered, 545
Commissioners in Lunacy, new, i
Consciousness, perceptive, 396
———— sensational, 230
Crime, endemic, nature of, 263
——— how affected by education, 275
———— the press, 277
——— juvenile, xxxvi
——— mental phenomena of, 428
——— statistics of, 659
Crimes, numerous in England, 243
Criminal insanity, liv
———— lunatics, by J. Russell Reynolds, M.D., lxxxvii
———— responsibility, by R. H. Semple, M.D., 305

D.

Damerow, Dr., on Monomania, 468
Delusions, epidemic form of, 262
Denham, Rev. J. F., on the connexion between morbid physical and religious phenomena, 39, 385
Diagnosis of diseases of the brain, by J. R. Reynolds, M.D., 90
Dove, William, 584
———— confession of, 650
Dreams, Dugald Stewart's theory of, 551
———— how produced, 380
———— and apparitions, physiological and psychological phenomena of, 373, 544
Dreamers, *egoism* of, 558
Dundee Royal Asylum, 189
Dunn, Robert, F.R.C.S., on physiological psychology, 226, 395

E.

Edinburgh Asylum described, 57
Education, its influence on crime, 275
Emperor of Russia, the late, his mental condition, xxxvii
Epidemics, moral and criminal, 240
Esquirol, Monsieur, on monomania, 518
Ethnological psychology, 171
Evidence, medical, on Palmer's trial, 429

F.

Fanaticism, by Dr. Franz, 473
Female intellect, its relation to the arts, 540
Foreign psychological literature, 101, 291, 465
France and Russia compared, 311, 315
Frank, Dr. Herman, death of, xv
Franz, Dr., on fanaticism, 473

G.

Gall, founder of physiological psychology, 411
German philosophical writers, 325
Glasgow Royal Asylum, 62
Goethe, Life of, by G. H. Lewes, vi
Goître and cretinism, 480

H.

Hagen, Dr. F. W., on the relations of anatomy and psychiatry, 465
Hallucinations considered, 509
Hamilton, Sir William, lxvi
Hearing, sense of, its effect on dreaming, 381
Hoppus, Professor, LL.D, psychology of Leibnitz, by, 325
——— on the psychology of Malebranche, 608
Human mind, letters on the philosophy of the, by Samuel Baily, xiii

I.

Idiocy, moral, 285
——— statistics of, 628
Infanticide, epidemic, 267
Insane, autobiography of the, 66, 202
——— chemistry of their urine, 488
Insanity, causes of, 303
——— ——— by M. Trelat, 479
——— criminal, liv
——— degrees of in Scotland, recognised, 55
——— doubtful, detection of, 291
——— in children, peculiar form of, 107
——— latent, how detected, 87
——— legal treatment of, xxiii
——— medical jurisprudence of, by Forbes Winslow, M.D., D.C.L., 143
——— plea of, 126
——— statistics of, 628
——— varieties of, 502
——— and dreaming, on the identity of, by M. Moreau, 102
Inspectors of Asylums in Scotland, how elected, 54
Intellect, how affected by old age, lxv
Intemperance, a type of disease, ii
Introspection, definition of, 385
——— evils arising from, 390
——— morbid religious phenomena attending it, 389
——— not the normal condition of the mind, 385
——— prevention and cure of, 393
Ireland, District Hospitals for the insane in, 642

L.

Leibnitz, psychology of, by Professor Hoppus, 325
——— character of his mind, 328
——— his writings, 329
——— his doctrine relating to human knowledge, 331
——— as a mathematician, 348
——— as a metaphysician, 349
——— and Locke compared, 337
Leuret, M., his writings on psychology, 412
Levison, J. L., on obscure nervous diseases, 643
Lewes, G. H., Life of Goethe, by, vi
Lindsay, W. Lauder, M.D., on the urine of the insane, 488
Literature of the quarter, vii
Locke and Leibnitz compared, 337
Lucid intervals, 143
Lunar influence, 144
——— in the production of bodily disease, 150
——— light, morbid phenomena of, 165
——— writers on, 150
Lunacy Commissioners, new, i
Lunatic Asylums in Scotland, Public, 51
——— leaves from the diary of a, 562
Lunatics, Chancery, the law relating to, 111
——— in North Britain, laws applicable to, 51

M.

Malebranche, Psychology of, by Professor Hoppus, 608
Man, mental faculties of, how divided, 377
Maniacs, tendency to murder in, lxxi.
Mankind, how divided, 177
Medical Officers of Asylums, Annual Meeting of, 644
Melancholy, defined, 504
Mental depression, considered, 89
——— disorder, unrecognised forms of, by Forbes Winslow, M.D., D.C.L., 82, 282
——— faculties, how manifested, 509
——— philosophy, the study of, iii.
Mind, disordered and insane, distinguished, 83
——— national, considered, 311
Monomania, 502
——— by Dr. Damerow, 468
——— its existence questioned, 505
——— medico-legal consultation, on a case of, by M. Baillarger, 101
Montrose Royal Asylum, 195
Moore, his last days, lxiii

Moral and criminal epidemies, 240
Moral idiocy, 285
Morbid phenomena of lunar light, 165
——— physical and moral, on the connexion between, 572
Morbid physical and religious phenomena, the connexion between, by the Rev. J. F. Denham, 39, 385
Mormonism, 260
Murray's Royal Asylum, 183
Murder, in groups, 265
——— tendency to, in maniacs, lxxi
——— wilful, considered, lxxviii
Muscular lassitude, induced during sleep, case of, 554
Mylne, Mr. Commissioner, death of, i.

N.

National characteristics, considered, 311
Nations, affinities of, how traced, 176
Nightmare, case of, from pressure on the chest, 548
Notoriety, love of, an incentive to crime, 273

O.

"Oligomania," 505

P.

Palmer, Wm., trial of, 419
——— medical evidence on, 429
Peace, considered, 311
Philosophical writers, German, 325
Philosophy of sleep, 374
Physical and religious phenomena, morbid, on the connexion between, by the Rev. J. F. Denham, 39, 385
Physiological psychology, by Robt. Dunn, 226, 395
Physiological and psychological phenomena of dreams and apparitions, 544
Poisoning, frequency of, 271
——— sporadic causes of, 269
Press, the, its effect on crime, 277
Psychiatry, practical observations on, 301
Psychological literature, foreign, 101, 291, 465
Psychology, ethnological, 171
——— of Leibnitz, by Professor Hoppus, 325
——— physiological, by Robt. Dunn, 226
Public Lunatic Asylums in Scotland, notes on, by John Webster, M.D., F.R.S., 183, 352
——— executions, effect of, xxix
Punishment, uncertainty of, conducive to crime, 280

R.

Remorse, definition of, 39
——— spurious, 40
——— morbid and physical origin of, 40
——— irrational nature of, 43
——— its prevention and cure, 49
Religious and physical phenomena, morbid, on the connexion between, by the Rev. J. F. Denham, 39, 385
Religious suicides, 447
Reynolds, J. Russell, M.D., on criminal lunatics, lxxxvii
——— on the diagnosis of the brain, 90
Russia and France, compared, 311, 315

S.

Sadleir, John, suicide of, xxxviii
Scotland, Inspectors of Asylums in, how elected, 54
——— Laws applicable to lunatics in, 51
——— Public Lunatic Asylums in, notes on, by John Webster, M.D., F.R.S., 51, 183, 352
Self mutilation, 268
Semple, R. H., M.D., on criminal responsibility, 305
Sensational consciousness, 230
Senses, the, effect of, on dreams, 380
——— special, 235
Sight, 237
Sleep and wakefulness, compared, 23
Sleep-vigil, phenomena of, 27
Sleep, philosophy of, 26
Smell, sense of, its effects on Dreams, 380
Social progress, xxxiv
Somnambulism, 22
——— condition of the brain in, 36
——— artificial, 38
——— phenomena of, 27
Somno-vigil, condition of the functions of the, 35
Soul and understanding, difference between, lxxvi
Spirit rapping, iii
——— Sir D. Brewster on, iii
——— somatic origin of, 261
Spiritualism, 260
Stewart, Dugald, his theory of Dreams, 375, 551
Statistics of insanity, 628
Suicide, a psychological question, 445
——— amongst children, 296

Suicide and suicidal insanity, by A. Brierre de Boismont, 445
——— from *ennui*, considered, 458
——— hereditary, 451
——— in women, 457
——— physiology and symptomatology of, 463
——— singular case of, xlvii, l, li
——— verdict in cases of, 452
Suicides, religious, 447
Suicidists in Paris, modes of death resorted to by, 461

T.

Tawny races, psychological characteristics of, 179
"Temper disease," 282
Ticket of leave men, meeting of, lxi
Touch, 235
Trelat, M., on the causes of insanity, 479
Turner, Dr., retirement of, i
Triune Man, a discourse delivered at the London Hospital Medical College, by Andrew Clark, M.D., 594

W.

War, cause of, 4
War, effects of, 11, 15, 321
—— pathologically considered, 17
Webster, John, M.D., F.R.S., on the Public Lunatic Asylums in Scotland, 51, 183, 352
Wilkes, Dr., Commissioner in Lunacy, ii
Winslow, Forbes, M.D., his opinion in Dove's case, 585
——— on medical jurisprudence of insanity, 143
——— on unrecognised forms of mental disorder, 82, 282
Witchcraft, 255
Woman, in her social relations, 521
——— her social position in England, 534
——— her social position among the Hebrews, 526
Women, ancient position of, among the Egyptians, 527
——— as nurses, 539
——— Catholic, their position, 538
——— influence of, 523
——— literary, 542
——— liberty of, in Sparta, 528

END OF VOL. IX.

